# Concomitance of Pericardial Tamponade and Pulmonary Embolism in an Invasive Mucinous Lung Adenocarcinoma with Atypical Presentation: Diagnostic and Therapeutic Pitfalls—Case Report and Literature Review

**DOI:** 10.3390/ijms25158413

**Published:** 2024-08-01

**Authors:** Nicoleta Sorina Bertici, Cristina Tudoran, Razvan Adrian Bertici, Ovidiu Fira-Mladinescu, Dragos Catalin Jianu, Caius Glad Streian, Raluca Elisabeta Staicu, Andrei Raul Manzur, Ana Lascu

**Affiliations:** 1 Department XIII, Pulmonology University Clinic, “Victor Babeș” University of Medicine and Pharmacy Timișoara, Eftimie Murgu Square No. 2, 300041 Timișoara, Romania; bertici.nicoleta@umft.ro (N.S.B.); mladinescu@umft.ro (O.F.-M.); 2 Center for Research and Innovation in Personalised Medicine of Respiratory Diseases, Pulmonology University Clinic, “Victor Babeș” University of Medicine and Pharmacy Timișoara, Eftimie Murgu Square No. 2, 300041 Timișoara, Romania; razvan.bertici@umft.ro; 3 IInd Pulmonology Ward, Clinical Hospital of Infectious Diseases and Pulmonology “Victor Babeș” Timișoara, Gheorghe Adam Street 13, 300310 Timișoara, Romania; 4 Department VII, Internal Medicine II, Discipline of Cardiology, “Victor Babeș” University of Medicine and Pharmacy Timișoara, Eftimie Murgu Square No. 2, 300041 Timișoara, Romania; 5 Center of Molecular Research in Nephrology and Vascular Disease, “Victor Babeș” University of Medicine and Pharmacy Timișoara, Eftimie Murgu Square No. 2, 300041 Timișoara, Romania; 6 Cardiology Clinic, County Emergency Hospital “Pius Brinzeu”, Liviu Rebreanu, No. 156, 300723 Timișoara, Romania; 7 Doctoral School Medicine-Pharmacy, “Victor Babeș” University of Medicine and Pharmacy Timișoara, Eftimie Murgu Square No. 2, 300041 Timișoara, Romania; raluca.staicu@umft.ro (R.E.S.); andrei.manzur@umft.ro (A.R.M.); 8 Department VIII, Clinic of Neurology I, “Victor Babeș” University of Medicine and Pharmacy Timișoara, Eftimie Murgu Square No. 2, 300041 Timișoara, Romania; jianu.dragos@umft.ro; 9 Centre for Cognitive Research in Neuropsychiatric Pathology (Neuropsy-Cog), Faculty of Medicine, “Victor Babeș” University of Medicine and Pharmacy Timișoara, Eftimie Murgu Square No. 2, 300041 Timișoara, Romania; 10 Department VI Cardiology-Cardiovascular Surgery, “Victor Babeș” University of Medicine and Pharmacy Timișoara, Eftimie Murgu Square No. 2, 300041 Timișoara, Romania; streian.caius@umft.ro; 11 Advanced Research Center of the Institute for Cardiovascular Diseases, “Victor Babeș” University of Medicine and Pharmacy Timișoara, Eftimie Murgu Square No. 2, 300041 Timișoara, Romania; 12 Institute for Cardiovascular Diseases of Timișoara, “Victor Babeș” University of Medicine and Pharmacy Timișoara, Gheorghe Adam Street, No. 13A, 300310 Timișoara, Romania; lascu.ana@umft.ro; 13 Department III Functional Sciences—Pathophysiology, “Victor Babeș” University of Medicine and Pharmacy Timișoara, Eftimie Murgu Square No. 2, 300041 Timișoara, Romania; 14 Centre for Translational Research and Systems Medicine, “Victor Babeș” University of Medicine and Pharmacy Timișoara, Eftimie Murgu Square No. 2, 300041 Timișoara, Romania

**Keywords:** invasive mucinous adenocarcinoma of the lungs, pericardial tamponade, pulmonary embolism, chest computed tomography

## Abstract

The invasive mucinous adenocarcinoma of the lungs (LIMA) is an uncommon histological subtype of the mucinous adenocarcinoma. In this article, we present the case of a patient with a very high cardiovascular risk profile, diagnosed with LIMA, pericardial tamponade due to secondary dissemination, and pulmonary embolism, whose management rouses many challenges. Despite receiving the correct anticoagulant and antiaggregant therapy, our patient developed repeated acute major cardiovascular events leading to a fatal outcome. To gather additional information on LIMA and the above cluster of pathologies, we performed the first research of the international medical literature for scientific articles published in the last eight years on PubMed, ResearchGate, Clarivate, and Google Scholar. As the first literature research failed to identify any case similar to our patient, we performed a second study of the same databases for subjects with lung adenocarcinoma instead of LIMA and the same comorbidities, and we found 10 cases. LIMA is a less frequent type of adenocarcinoma, with polymorphic radiologic appearances on the chest computed tomography, frequently mimicking pneumonia, and thus delaying the diagnosis and therapy. It has a worse prognosis and higher mortality than the common adenocarcinoma, but information on its secondary dissemination and complications is still required.

## 1. Introduction

The invasive mucinous adenocarcinoma of the lung (LIMA) represents a histological subtype of the mucinous adenocarcinoma previously referred to as mucinous bronchioloalveolar carcinoma [1]. LIMA is relatively uncommon, representing about 2% to 10% of all lung adenocarcinomas. This peculiar form was reclassified in the 2015 WHO’s classification of lung tumors, and this entity was adopted by the International Association for the Study of Lung Cancer [1,2]. The mucinous adenocarcinomas represent a morphologically distinct group of lung adenocarcinomas. They were divided into mucinous adenocarcinoma in situ, LIMA, and colloidal adenocarcinoma. Of these, LIMA is the most common and was formerly classified as mucinous bronchioloalveolar carcinoma, where tumor cells have abundant intracytoplasmic mucin organized in a goblet or columnar morphology [3]. Mucinous material with macrophages and tumor nests floating in the alveolar space, as seen in this carcinoma, tends to be more diffuse. Distinguishing molecular features further supports the classification of these tumors into separate diagnostic categories. The classification into separate variants is based on morphology, immunoprofile, and genomic and clinical characteristics [4]. Due to its more aggressive behavior when compared to non-mucinous bronchioloalveolar adenocarcinoma [5], LIMA’s name was completed with the term “invasive”.

The most frequent symptoms and signs are cough, sputum, dyspnea, weight loss, hemoptysis, and fever, but more than half of all patients with bronchioloalveolar carcinoma are asymptomatic [5]. On a chest computed tomography (CCT), it can appear as an isolated single nodule, segmental or lobar consolidation, or diffuse nodules, but also as heterogeneous ground-glass opacity mimicking various types of pneumonia [3]. Compared with other subtypes, LIMA also extends by contiguity and through the lymphatic ways, but it frequently demonstrates an increased tendency of bronchogenic spreading through bronchial airways, representing an additional independent poor prognostic predictor [6].

There is little information concerning LIMA’s tendency to secondary dissemination. While metastatic pleurisy has been described occasionally, we failed to find mention of pericardial dissemination, especially with tamponade.

LIMA is rarely diagnosed on CCT-guided needle biopsies due to insufficient tissue. Generally, when diagnosed, patients present a more advanced stage of disease, with an increased mortality rate and reduced overall survival [5,7].

With the recent advancements in genetic testing, several genetic disturbances, such as mutations in the Kirsten rat sarcoma viral oncogene homolog (KRAS), neuregulin-1 (NRG1) fusions, and rearrangements of the anaplastic lymphoma kinase (ALK), have been associated with LIMA, opening a new perspective for its diagnosis and treatment by interfering with these gene mutations and signaling pathways. On the other hand, the frequency of epidermal growth factor receptor (EGFR) mutations, ALK, and ROS1 translocations are usually lower or even negative compared with other subtypes of adenocarcinomas, thus rendering them ineligible for tyrosine kinase, so their response to chemotherapy is modest [5,7,8,9]. Of course, the presence of complications and associated comorbidities impairs patients’ therapeutic approach and their prognosis even more.

In our study, we debated over a case of LIMA with bilateral pleurisy and pericardial tamponade with sero-hemorrhagic exudate due to secondary dissemination, diagnosed in a patient with very high cardiovascular risk who was diagnosed concomitantly with PE and subsequently with significant monovascular coronary artery disease and whose diagnostic and therapy rose multiple challenges. We concomitantly studied the medical literature for available data to learn about the most recent research concerning LIMA and, secondly, to find out whether there were other cases reporting similar pathological associations.

## 2. Results

A 52 year–old man who was a heavy smoker (30 pack-years), without other toxic or noxious exposure or significant family history, diagnosed with diabetes mellitus type 2 and treated with oral glucose-lowering therapy (Metformin 500 mg twice daily), hyperlipidemia and obesity grade 1 (body mass index = 31.67 kg/m^2^) was admitted in the emergency room on 30 September 2023 with suddenly installed acute severe inspiratory dyspnea, chest pain, marked fatigability, and anxiety. The medical exam revealed facial and peripheral cyanosis, jugular venous distension, signs of cardiogenic shock with low blood pressure (60/45 mmHg), a heart rate of 112 beats per minute, muffled heart sounds, 34 breaths/minute, and reduced spontaneous oxygen saturation on peripheral pulsoximetry (70%). On the electrocardiogram (ECG), there was evidence of sinus tachycardia and micro voltage.

**Figure 1:**
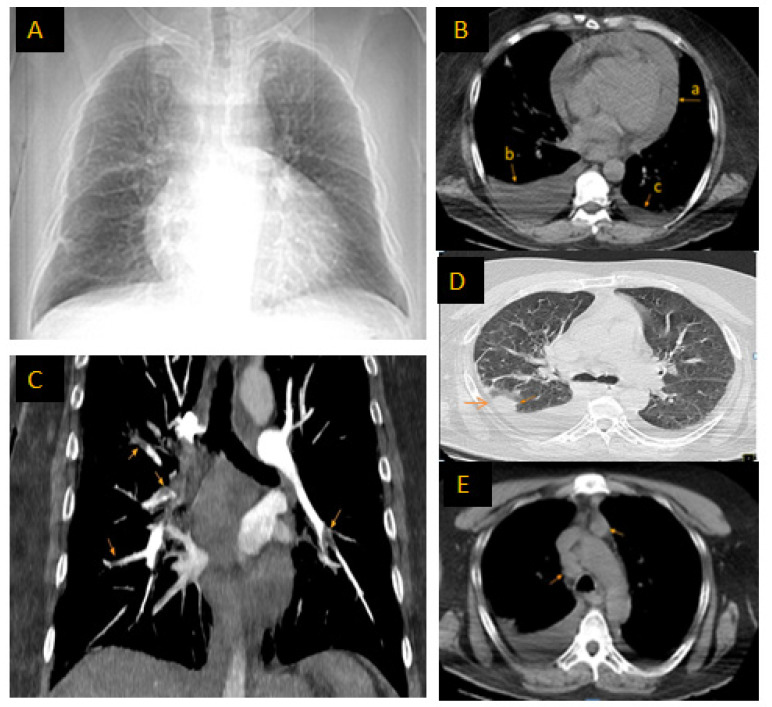
Chest computed thorax angiography performed at admission: (**A**): chest frontal view with global cardiomegaly due to pericarditis and enlarged mediastinum; (**B**): section at the level of the heart: (a) circumferential pericarditis; (b) bilateral pleurisy, more important on the right side; and (c) minimal effusion on the left side; (**C**): multiple bilateral filling defects central, segmental, and subsegmental, suggesting both acute and chronic PE; (**D**): ground-glass attenuation with small consolidation areas in the right medium lobe and mixed emphysematous dystrophy; and (**E**): mediastinal polymacroadenopathy.

The transthoracic echocardiography (TTE), performed in the emergency room, revealed the presence of a circumferential pericardial effusion of 3.1 cm, a “swinging heart”, and telediastolic collapse of the right atrium. CCT-angiography (CCTA), performed with the administration of intravenous contrast material, revealed circumferential pericarditis, bilateral pleural effusion (more pronounced on the right side), multiple bilateral filling defects located centrally, segmentally, and subsegmentally, with an aspect suggesting acute pulmonary embolism (PE), as well as signs of a preexisting PE, mixed emphysematous dystrophy, and mediastinal polymacroadenopathy, as shown in Figure 1.

Laboratory tests revealed highly elevated levels of the D-dimers and of the NT-proBNP level, inflammatory syndrome, elevation of aspartate and alanine aminotransferase (ASAT, ALAT), and hyperglycemia.

A pleuropericardial window was urgently performed with the evacuation of 850 mL of a sero-hemorrhagic pericardial fluid with the following characteristics: protein level = 5.808 g/dL, LDH = 3757 IU/L, glucose = 42 mg/dL) with cytology positive for adenocarcinoma. Concomitantly, 500 mL of a serocitrine pleural fluid (transudate) was evacuated from the right hemithorax. Retrocardiac and right pleural drain tubes were mounted and connected to an aspiration drainage system for two days. A few days after the emergency surgical intervention for cardiac tamponade, it was decided to perform angiocoronarography, considering the patient’s intense chest pain at admission and his very high cardiovascular risk profile. This investigation revealed a monovascular coronary artery disease (70% stenosis in the middle segment of the left anterior descending artery (LAD)), but the stenting of the LAD lesion was delayed considering the decrease of troponin levels and the alleviation of chest pain. Anticoagulant therapy with unfractionated heparin, under the strict control of activated partial thromboplastin clotting time (APTT), and antiplatelet treatment (Aspirin) were initiated with caution, considering the presence of the sero-hemorrhagic pericardial effusion, gastroprotection, and supplemental oxygen therapy. A second TTE performed the next day determined dilated left atrium, dilated right ventricle, and left heart concentric hypertrophy with an ejection fraction of 50%, a moderate-to-severe estimated systolic pulmonary artery pressure (PAP) of 60 mmHg. Right heart catheterization was performed to evaluate the hemodynamics of pulmonary circulation. The results showed only mild precapillary pulmonary hypertension with systolic and diastolic PAPs (PAPs and PAPd, respectively) of PAPs/PAPd = 29/14 mmHg, a mean PAP = 20 mmHg, pulmonary capillary wedge pressure (PCW) of 11 mmHg, and pulmonary vascular resistance (PVR) of 3.28 mmHg/L/min. Furthermore, a dilated right ventricle with mild wall hypertrophy was determined. Peripheral Doppler vascular echography evidenced a dilated superficial venous system, with stasis and indirect signs suggesting a right popliteal vein thrombosis. After seven days, the patient’s clinical condition improved significantly, and his vital signs normalized.

Considering the associated pulmonary pathology and the high suspicion of a pulmonary adenocarcinoma, the patient was transferred to the pneumology clinic with the following diagnosis: cardiac tamponade due to a sero-hemorrhagic pericardial effusion of probable neoplastic etiology (metastasis of a lung adenocarcinoma); acute PE (possibly with repeated previous PE); mild secondary pulmonary hypertension; severe functional tricuspid insufficiency; monovascular coronary artery disease; mild degenerative mitral insufficiency; chronic heart failure with preserved ejection fraction; right popliteal venous thrombosis; chronic obstructive pulmonary disease mild form; obstructive sleep apnea syndrome, with an apnea–hypopnea index of 25.1 per hour classifying it as moderate form; type II diabetes treated with oral antidiabetic drugs; obesity grade I; and combined hyperlipidemia. The anticoagulant treatment was continued with apixaban 5 mg twice daily until discharge according to guidelines recommendations, aspirin (75 mg/day) associated with diuretics (spironolactone/furosemide 50/20 mg/day), beta-blockers (bisoprolol 2.5 mg/day), statins (atorvastatin 80 mg/day), and sodium-glucose co-transportor 2 (SGLT2) inhibitors (dapagliflozin). Further investigations aimed to identify the primary neoplasia’s location. In this regard, after ten days, a second CCTA was performed, indicating the persistence of bilateral filling defects (proximal and distal lower lobes, proximal lingular, and lateral segment of the middle lobe). Ground-glass areas with a focal and random character were distributed in the upper left lung field, most likely due to perfusion disorders. Furthermore, regression of the pleural effusion and pericarditis, mixed emphysematous dystrophy, and enlarged mediastinal polymacroadenopathy were detected. Multiple investigations (bronchoscopy, gastroscopy, and colonoscopy and abdominal and pelvis computed tomography) failed to determine a precise location of the primary neoplasm. Also, collagenosis, sarcoidosis, vasculitis, and infectious pathologies (including HIV and tuberculosis) were excluded. The genetic profile of the thrombophilia risk revealed the heterozygous MTHFR A1298C variant, PAI-1 4G/4G homozygous genotype, and EPCR with the presence of A2/A2 alleles (indicating decreased fibrinolytic activity and favoring thrombosis). Thus, associated risk factors for PE were confirmed. The patient’s evolution was stable, with a good condition and mild clinical symptoms (only effort dyspnea, anxiety, and weight loss of about 8 kg within a hypocaloric diet context) and without recurrence of pericarditis or pleurisy.

After one month, the patient presented sudden dysarthria, ataxic left hemiparesis with a pseudoradial aspect of the left hand, and inability to walk and stand upright. Although the patient was under therapy with apixaban 5 mg twice daily and aspirin 75 mg daily, he interrupted it for approximately 48 h due to a dental procedure. He was admitted to the neurology department of his hometown, where an acute right sylvian and left cerebellar ischemic vascular stroke, without excluding possible associated thromboembolism, was confirmed by cerebral magnetic resonance imaging (MRI). The cessation of both anticoagulant therapy and aspirin was considered responsible for the stroke by his treating physician, which is the reason why no anti-Xa activity test or other hemostasis tests were considered necessary at that time. The third CCTA was performed with a stationary image compared with the previous evaluations. The motor deficits are partially remitted after treatment with neurotrophic drugs, statin, and associated physical therapy. After two months, the fourth chest CCTA was performed and revealed the absence of central, lobar, and segmental vascular filling defects of embolic type, except a partial filling defect in the projection of the posterior segmentation of the left lower lobe; the mediastinal polymacroadenopatia increased in dimensions compared with previous exams, and the ground-glass lesions persisted.

**Figure 2:**
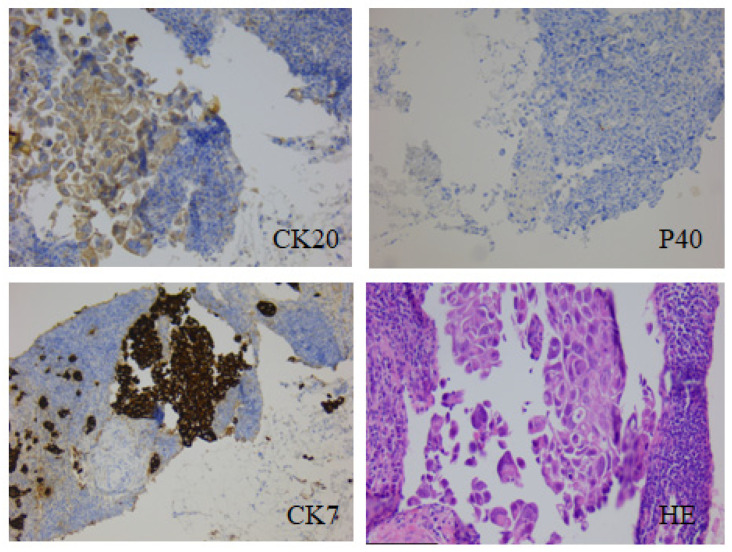
Immunohistochemical examination/optical microscopy: Analysis in the IHC context reveals a metastatic carcinomatous proliferation with an immunoprofile compatible with a mucinous, poorly differentiated G3 adenocarcinoma, most likely of pulmonary origin. TTF1 positive, CK7—cytokeratin 7 intensely positive, CK20—cytokeratin 20 positive low intensity, LCA negative, CDX2 negative, P40—squamous cells carcinoma specific marker negative. Histopathological diagnosis: Those described correspond to a non-microcellular carcinoma with an immunoprofile compatible with a mucinous bronchoalveolar adenocarcinoma M8253/3. Microscope magnification: CK20—124 µm; P40, CK7—248 µm; HE—119 µm.

The repeated bronchoscopy evidenced extrinsic bronchial compression due to the mediastinal adenopathy, but the transbronchial approach was not possible, and a transthoracic CCT-guided needle biopsy was postponed due to increased hemorrhagic risk versus thrombotic hazard considering the recent PE and anticoagulant therapy but also to the uncertainty of obtaining a suitable biopsy material. D-dimers maintained their elevated levels throughout this period (11.04 mg/L–6.74 mg/L–8.13 mg/L, while the normal value must be below 0.50 mg/L). The tumor markers were also determined, of which carbohydrate antigen 19-9 (CA19-9) initially was 533.53 U/mL, which significantly increased to 1253.66 U/mL during two months. The diagnosis “key” was a left-sided supraclavicular adenopathy, which was biopsied. The cytology exam reveals malignant tumor proliferation in favor of a poorly differentiated adenocarcinoma. Analysis in immunohistochemistry context revealed a metastatic adenocarcinomatous proliferation with an immunoprofile compatible with a poorly differentiated G3 mucinous adenocarcinoma, most likely of pulmonary origin, with positive thyroid transcription factor 1 (TTF1), intensely positive LIMA’s typically expressed cytokeratin 7 (CK7) (88–94.7%), cytokeratin 20 (CK20) positive at low intensity (79%), negative leukocyte common antigen (LCA) negative, negative caudal type homeobox 2 (CDX2), and negative squamous cell carcinoma specific marker (P40) (Figure 2) [7,8,10].

The final histopathological diagnosis concluded that the description corresponded to a non-microcellular carcinoma with a LIMA-compatible immunoprofile M8253/3. Thus, unexpectedly, immunohistochemistry tests point toward LIMA; meanwhile, the patient was without respiratory symptoms, tumor markers suggestive of adenocarcinoma, and four lung CCT angiographies, and two bronchoscopies could not localize its precise position to perform a proper biopsy. The patient was scheduled to perform a positron-emission tomography to determine the exact location of the lung tumor, and, subsequently, after a CCT-guided biopsy, the oncological treatment was to be initiated. Unfortunately, shortly after this last evaluation, less than three months after the first clinical presentation, the patient suffered a new massive stroke (possibly due to a new embolism) in the territory tributary to the junction of the left median carotid artery and the deep carotid artery, followed by hemorrhagic transformation, which resulted in death within four days.

## 3. Discussion

According to epidemiological studies and medical literature data, LIMA is the rarest type of lung adenocarcinoma, with a low incidence rate (it comprises 1.2% of all lung cancers, respectively between 2 and 9.3% of all lung mucinous adenocarcinomas) [2] and a worse prognosis and contradictory survival rates compared with non-mucinous adenocarcinomas [11]. Matsui et al. determined that although there is no significant difference regarding the 5-year survival rates between LIMA and other adenocarcinomas, those with LIMA and intrapulmonary recurrence had a worse outcome [12].

Starting from our case report with multiple diagnostic and therapeutic challenges and considering the scarce available data, we performed two pieces of research on the medical literature published in the last 8 years, available on Clarivate, PubMed, ResearchGate, and Google Scholar, the first aiming at finding out case presentations with LIMA with similar diagnostic challenges and the second aiming at identifying case reports debating on similar secondary dissemination, complications, and comorbidities that deeply influenced patient’s prognosis by delaying an accurate diagnosis and impacting an adequate therapy.

To analyze possible analogies between our case and other case presentations, in the four researched databases, we identified seven case reports with LIMA among all the articles published in the last 8 years—as described in Table 1. We also discovered 11 original articles and two systematic reviews focusing more on the diagnostic, evolution, treatment, and outcome of patients with LIMA than on clinical presentations [4,5,7,9,11,12,13,14,15,16,17,18,19]. The majority of these original articles assessed the possibility of predicting the outcome based on CCT findings and determining a prediction model for the evolution of the disease [7,9,11,14,15,16,17,18,19], while others compared the prognostic of LIMA to the adenocarcinoma of the lung and concluded that the first pathology has a worse evolution [11,12]. The more advanced age of the patients, the aspect of LIMA on CCT, and the associated dissemination were negative predictive factors [7,15,16]. It is worth mentioning that in most cases, LIMA does not have a characteristic, distinct aspect of the CCT, and it can be easily confused with pneumonia. Sometimes, ground-glass lesions appear on the CCT, as described by several authors, indicating a worse prognosis [7,9,15,16,17,18,19].

We detected seven case reports of patients with LIMA, predominantly older men (over 66 years old); see Table 1. The most frequent aspects of LIMA, as described on the CCT, were nodules and masses, and only one patient had ground-glass lesions, as did our patient [20]. Only two patients had pleural effusion [20,21], and two subjects had mediastinal adenopathies [22,23]. We did not find in the researched medical literature any cases with pericardial tamponade in LIMA or association with PE.

**Table 1:** Case presentations of patients with LIMA in the last 10 years.

Authors/Year	No. of Patients/Gender/Age	Description on CCT	Secondary Dissemination
Charleston et al./2023 [24]	1/M/88 years	A dense consolidation with a cavitary lesion in the left lower lobe	Not described
Yang et al./2021 [22]	1/F/68 years	Multiple nodules of different sizes and mass-like soft tissue density in the left upper lobe, lower lobe, and subpleural region	Mediastinal and left hilum adenopathy,
Frick et al./2021 [25]	1/M/68 years	A bullous malformation in the right lower lobe with recurrent infection	Not described
Nakamura et al./2021 [26]	1/F/78 years	Multiple pulmonary nodules and cavities	Not described
Aoki et al./2020 [21]	1/M/77 years	Subpleural nodular opacities	Chilotorax, irregular pleural thickening, right pleural effusion
Anwar et al./2020 [23]	1/F/57 years	Right-sided lung mass	Mediastinal and cervical lymphadenopathy, cerebellar metastasis, adrenal gland metastasis
Masuzawa et al./2016 [20]	1/M/75 years	Thin-walled chest and ground-glass opacity	Right side pleural effusion

As we failed to identify any other case report in the researched medical literature presenting a patient with cardiac tamponade due to secondary dissemination as the first manifestation of LIMA, concomitantly with the inauspicious association of a PE that greatly complicated the patient’s management and, additionally, a very high cardiovascular risk profile resulting in acute coronary syndrome and ischemic stroke, we searched for case reports of individuals with pulmonary adenocarcinoma, cardiac tamponade, and PE/deep vein thrombosis. Massive pleural effusion, cardiac tamponade, and sometimes PE are complications often encountered in pulmonary malignancy, but it is exceptionally rare for these pathologies to occur simultaneously. We found nine case reports debating over 10 patients, three males and seven women, with lung adenocarcinoma and similarly associated pathology, as presented in Table 2.

Although, as shown in Table 2, female gender prevailed, we did not find in the medical literature specific data regarding a possible sex-related predisposition for this association of pathologies, but this may be due to the rarity of the combination of these three diseases in one patient and to the few case reports available. Taken individually, it was determined that PE has a higher incidence in women [27], while lung adenocarcinoma is also more often encountered in female patients [28], and a study on 216 million hospital admissions for tamponade revealed that 46.5% were females [29].

**Table 2:** Pulmonary adenocarcinoma with pericardial tamponade and PE.

Authors /Year	No. of Patients /Gender/Age	Adenocarcinoma Type	Aspect on CCT	Secondary Dissemination	Associated CCT Findings	Clinical Presentation	Associated Risk Factors
El Rahalete et al./2023 [30]	1/F/38 years	Moderately differentiated adenocarcinoma of the lung	Parenchymal lymph node of the upper lung lobe	Hepatic metastasis	Deep vein thrombosis of the left lower limb, pericardial tamponade, bilateral PE, bilateral pulmonary effusion	Respiratory distress, chest pain	Twin pregnancy
Itakura et al./2023 [31]	1/F/42 years	Lung adenocarcinoma	Not described	Not described	PE, pericardial effusion	Dyspnea and tachycardia	Trousseau’s syndrome
Rahman et al./2021 [32]	1/M/54 years	Anaplastic lymphoma kinase-positive adenocarcinoma of the lung.	Not described	Vertebral and trochanteric metastasis, retroperitoneal and mediastinal lymphadenopathy	Pericardial tamponade, bilateral deep vein thrombosis, PE	Dyspnea	Acute COVID-19 infection
Li et al./2019 [33]	1/M/65 years	Adenocarcinoma	Multiple bilateral pulmonary nodules	Not described	Pleural effusion, pericardial tamponade, massive PE	Dyspnea	Persistent cough
Huang et al./2019 [34]	1/F/48 years	Adenocarcinoma	Right upper lung lobe mass	Not described	Massive PE, pleural effusion and cardiac tamponade	Syncope and dyspnea	Not described
Pazooki et al./2019 [35]	1/M/50 years	Lung adenocarcinoma	Not described	Not described	Bilateral emboli in left and right pulmonary arteries, moderate pericardial effusion	Dyspnea	Chemotherapy
Mufti et al./2018 [36]	1/F/63 years	Adenocarcinoma with BRAF mutation	A nodule in the right upper lobe	Mediastinal adenopathy	Bilateral PE, large pericardial effusion	Dyspnea, palpitations, chest pain	Not described
	1/F/66 years		Right upper lobe necrotic mass	Mediastinal adenopathy and bilateral adrenal masses	Pericardial effusion, and right pleural effusion	Dyspnea, cough, fatigue, weight loss	Not described
Kandasamy et al./2015 [37]	1/F/40 years	primary moderately differentiated adenocarcinoma of the lung	Soft tissue focus in the apico-posterior segment of the left lung upper lobe	Lymph nodes in the mediastinum, bony metastasis	Large pericardial effusion, right PE	Dyspnea, palpitations	Not described
Akhbour et al./2014 [38]	1/F/63 years	Moderate differentiate adenocarcinoma	Mass lesion in the right lower lung lobe (2.1 cm)	Not described	PE and cardiac tamponade	Dyspnea, chest pain	Not described

In all those cases, the cardiac tamponade was supposed to represent a secondary dissemination of the adenocarcinoma, especially as in most cases, it was accompanied by pleural collections, and the analysis of the pleural/pericardial exudate revealed neoplastic cells, as in the case of our patient. As for the etiology of the associated PE [31,33], consider that it was due to Trousseau’s syndrome, a pathological state identified sometimes in patients with malignancy and characterized by spontaneous recurrent or migratory venous thromboses and/or arterial emboli due to a hypercoagulability syndrome associated with cancer. Initially, we also took into consideration this syndrome in our patient, but further investigations, such as genetic testing for thrombophilia indicating a decreased fibrinolytic activity and favoring thrombosis and the evidence of recurrent PEs on the CCT angiogram, rolled out this supposition.

All authors underlined the enormous provocation represented by the necessity of administering anticoagulants to treat PE, according to guidelines, in patients with a high bleeding risk and a formal contraindication to this therapy due to the neoplasia and to pericarditis where anticoagulants should be avoided, and even more if the pericardial fluid has a hemorrhagic appearance [33]. As in other conditions with increased bleeding hazard but with an absolute indication for anticoagulation, this medication should be given with caution, under strict monitoring, and at the lowest therapeutic dose [35,39].

Regarding anticoagulant therapy in patients with venous thromboembolism, particularly in those with cancer, the European Society of Cardiology guidelines [40] and a large meta-analysis of nine randomized controlled trials (5443 patients) demonstrated that therapy with low-molecular weight heparins is beneficial for patients with lung cancer, and moreover, thrombo-prophylaxis with this drugs reduced the risk for VTE (RR 0.54, 95% CI: 0.43–0.69) without an increase in overall survival (1.02, 95% CI: 0.83–1.26) in this population [41].

In all debated cases, pericardiocentesis/pericardial window was performed to save the patient’s life. In an original article by [42], by analyzing 1,207,580 patients with lung cancer—of whom 0.6% had pericardial tamponade, most of them men—treated with chemotherapy, it was found that pericardiocentesis is mandatory to restore the patient’s hemodynamic, but it triggers a worse prognosis with reduced survival. All authors complain about the difficulty of performing a pericardial fluid evacuation in a patient receiving anticoagulant therapy. Perhaps the most dramatic case of those presented above was that reported by [30] relating to a 38-year-old patient with a twin pregnancy of 16 weeks who was diagnosed with deep vein thrombosis of the left lower limb. She was treated with a therapeutic dose of low molecular weight heparin and subsequently developed a PE despite this medication.

Most of the patients described in these case reports had a worse prognosis and died shortly after the pericardial drainage or during further diagnostic procedures. In a few, it was possible to initiate chemotherapy, but their survival was only for a few months.

Our case is particularly special because of the inauspicious association of pericardial tamponade, PE, deep vein thrombosis, the difficulties encountered in establishing his diagnosis, and even more by the unfavorable concomitance of coronary and cerebral artery disease, which determined a fatal course of the patient’s life. We failed to identify another similar case in the researched medical literature with such a cluster of severe pathologies and who developed several major cardiovascular events despite correct anticoagulant and antiaggregant therapy, determining, in the end, his death before initiating any chemotherapy for his cancer.

Our case report has several clinical implications. It is recommended to perform genetic tests for thrombophilia, especially in younger patients with PE and deep vein thrombosis (DVT) of questionable etiology. Our case presentation emphasizes the need for maintaining a consequent anticoagulation therapy for an adequate period of time in patients with venous thromboembolism and suspicion of lung cancer and to inform them and their family on the risk of interrupting this medication without a previous discussion with the treating cardiologist. We suggest performing anti-Xa activity and other hemostasis tests in patients on anticoagulant therapy and repeated venous and arterial embolic events. Our findings point out the importance of investigating comprehensively persistent atypical lung lesions (ground-glass), especially when associated with thromboembolic events since they could indicate lung cancer. In such cases, the biopsy of peripheral adenopathies could confirm the diagnosis.

The main limitation of our research is that we did not perform a systematic review of the medical literature, restraining our investigation to four databases. On the other hand, as we were not able to find any case with LIMA and this combination of pathologies, we researched some cases with lung adenocarcinomas and similar complications.

## 4. Materials and Methods

To identify articles debating over clinical cases of patients with LIMAs similar to our subject that would be suitable for a literature review, we performed the first research of the international medical literature for scientific articles published in English in the last eight years in PubMed, ResearchGate, Clarivate, and Google Scholar. We researched each database individually by using the following keywords: “lung invasive mucinous adenocarcinoma, “pericardial tamponade”, “pulmonary embolism”, “deep vein thrombosis”, “adenocarcinoma of the lungs”, and “secondary dissemination of LIMA”. All articles were introduced into Zotero, and duplicates were eliminated.

As we found several original articles performed on larger populations and two literature reviews on the diagnosis, prognosis, and therapy of LIMA, we employed them for the introductive part and, to a lesser extent, in the Discussion section. In the second part of our review, because we were able to identify any case presentations with LIMA and this cluster of pathologies (LIMA, pericardial tamponade, and PE/deep vein thrombosis), we performed a second study of the same databases for patients with lung adenocarcinoma, as a surrogate for LIMA, and the same associations, and we identified 10 case reports debating on patients with a similar combination.

## 5. Conclusions

LIMA represents an infrequent histological subtype of mucinous adenocarcinoma. Its diagnosis is difficult due to the multiple radiologic appearances on chest computed tomography, which frequently mimick pneumonia and thus delay a proper diagnosis. It is considered that it has a worse prognosis and higher mortality than common adenocarcinoma, but additional information on its secondary dissemination and complications is still required.
